# Predictors of positive response to cardiac resynchronization therapy

**DOI:** 10.1186/1471-2261-14-55

**Published:** 2014-04-29

**Authors:** Diana Rinkuniene, Silvija Bucyte, Kristina Ceseviciute, Silvijus Abramavicius, Kristina Baronaite-Dudoniene, Jolanta Laukaitiene, Tomas Kazakevicius, Vytautas Zabiela, Vytautas Sileikis, Aras Puodziukynas, Renaldas Jurkevicius

**Affiliations:** 1 Lithuanian University of Health Sciences, Kaunas, Lithuania; 2 Hospital of Lithuanian University of Health Sciences Kaunas Clinics, Kaunas, Lithuania; 3 Department of Cardiology, Lithuanian University of Health Sciences, Kaunas Clinics, Eiveniu 2, LT-50009 Kaunas, Lithuania

**Keywords:** Cardiac resynchronization therapy, Heart failure, Response

## Abstract

**Background:**

Approximately 30% of patients treated with cardiac resynchronization therapy (CRT) do not achieve favourable response. The purpose of the present study was to identify echocardiographic and clinical predictors of a positive response to CRT.

**Methods:**

The study included 82 consecutive heart failure (HF) patients in New York Heart Association (NYHA) functional class III or IV with left bundle branch block (LBBB), QRS duration ≥ 120 ms and left ventricular ejection fraction (LVEF) ≤ 35%. Statistical analysis was performed using IBM SPSS statistical software (SPSS v.21.0 for Mac OS X). A p value < 0.05 was considered statistically significant.

**Results:**

Echocardiographic response was established in 81.6% and clinical response was achieved in 82.9% of patients. Significant univariate predictors of favourable echocardiographic response after 12 months were smaller left ventricular end-diastolic diameter (LVEDD) (odds ratio [OR] 0.89; 95% confidence interval [CI] 0.82 - 0.97, p = 0.01), and smaller left ventricular end-systolic diameter (LVESD) (OR 0.91; 95% CI 0.85 - 0.98, p = 0.01). Lower uric acid concentration was associated with better echocardiographic response (OR 0.99; 95% CI 0.99 - 1.0, p = 0.01). Non-ischemic HF etiology (OR 4.89; 95% CI 1.39 - 17.15, p = 0.01) independently predicted positive clinical response. Multiple stepwise regression analysis demonstrated that LVEDD lower than 75 mm (OR 5.60; 95% confidence interval [CI] 1.36 - 18.61, p = 0.01) was the strongest independent predictor of favourable echocardiographic response.

**Conclusions:**

Smaller left ventricular end-diastolic and end-systolic diameters and lower serum uric acid concentration were associated with better response to CRT. Left ventricular end-diastolic diameter and non-ischemic heart failure etiology were the strongest independent predictors of positive response to CRT.

## Background

Approximately 1 – 2% of the adult population in developed countries have HF, and its prevalence rises to ≥ 10% in individuals 70 years of age or older [1]. Coronary artery disease (CAD) is the cause of approximately two-thirds of cases of systolic HF, although in many cases hypertension and diabetes are likely contributing factors [2].

HF is associated with substantial mortality and morbidity, and remains the most common hospital discharge diagnosis in elderly patients. Development of HF is characterised by progressive left ventricular (LV) remodelling, that further impedes LVEF and is responsible for progression of clinical symptoms. Results from mechanistic studies, observational evaluations and randomised controlled trials consistently demonstrated significant improvement in quality of life, functional status, and exercise capacity in HF patients in NYHA class III and IV who were assigned to active CRT. However, CRT does not provide any benefit to approximately 30% of patients [3-5]. Lack of response to CRT in these studies may be in part attributed to inappropriate echocardiographic and/or electrocardiographic criteria used to select patients for CRT. The purpose of our study was to identify initial echocardiographic and clinical parameters that predict positive response to CRT.

## Methods

### Patient population

The study included 82 consecutive HF patients, who underwent CRT implantation at Cardiology Department at Hospital of Lithuanian University of Health Sciences Kaunas Clinics, between January 2009 and December 2011. All patients met the following inclusion criteria: NYHA class III or IV despite optimal medical therapy, LBBB, QRS width ≥ 120 ms and LVEF ≤ 35%. Patients with previously implanted pacemaker or defibrillator, recent myocardial infarction or coronary artery bypass graft surgery (≤6 months), or decompensated HF were excluded.

Ischemic cardiomyopathy (ICMP) was diagnosed in patients with previous myocardial infarction, or coronary artery bypass graft surgery, or percutaneous coronary intervention (balloon and/or stent angioplasty), or angiografically documented significant coronary artery disease and history of angina pectoris.

All patients were in sinus rhythm, electrocardiogram at rest was recorded (measured on surface electrocardiogram leads, at a paper speed of 25 mm/s) and QRS duration was measured at baseline and after 12 months post CRT implantation.

Clinical evaluation and two-dimensional echocardiography were performed before CRT device implantation and repeated at 12 months of follow-up. Clinical evaluation included assessment of NYHA class and performance of 6 minute walk test (6-MWT). At 12 months of follow-up, patients, who achieved improvement in NYHA of at least 1 class and a ≥ 15% increase in 6-MWT, were classified as clinical responders.

Before the study all patients signed informed consent approved by Local Ethics Committee (Kaunas Regional Biomedical Research Ethics Committee, ref. n. BE-2-54).

Blood samples for uric acid concentration were taken one to two days before CRT implantation and were analysed in the laboratory of Lithuanian University of Health Sciences Hospital Kaunas Clinics.

### Two-dimensional echocardiography

Echocardiography was performed in all patients at rest in the lateral decubitus position, at baseline before device implantation and was repeated at 12-months follow-up. Transthoracic Doppler echocardiography was performed using a GE Vivid 7 system (GE Vingmed Ultrasound AS N-3190, Horten, Norway) with an M4S transducer. Standard transthoracic echocardiographic measurements were performed according to the Guidelines of the American Society of Echocardiography [6]. A standard evaluation of LV volumes was performed, and LVEF was calculated according to the Simpson’s equation. To minimize variability of measurements, all echo/Doppler evaluations were performed and analysed by the same physician, also the same transducer position and sample volume location were maintained throughout the recordings. All images were digitally stored for off-line analysis (EchoPac V.6.0.0; GE Vingmed).

Echocardiographic response was defined as an increase in LVEF of ≥ 5% and decrease of left ventricular end-systolic volume (LVESV) and left ventricular end-diastolic volume (LVEDV) by ≥ 15%.

### Device implantation

CRT implantation was performed through the subclavian or axillary vein access. Coronary sinus venogram was obtained using balloon catheter. LV lead was inserted into the posterolateral vein, which was selected according to anatomical characteristics of the vessels enter the appropriate electrode. The optimal position of the LV lead was defined according to LV lead impedance, threshold and no nervus phrenicus stimulation. The right atrial lead was conventionally positioned in the right atrium appendage and right ventricular lead in to the ventricular septum or right ventricular apex. Finally, all leads were connected to a dual chamber biventricular CRT device. The optimal atrioventricular (AV) delay was determined during simultaneous biventricular pacing by the simplified mitral inflow method: a long AV interval (200 ms) was programmed and gradually reduced by 20 ms, until A-wave truncation was observed.

### Statistical analysis

Statistical analysis was performed using IBM SPSS statistical software (SPSS v.21.0 for Mac OS X). Normally distributed continuous variables were presented as mean ± SD and were compared using Student *t*-test for paired and unpaired data. Statistical significance of differences between groups was analysed by Mann–Whitney *U* test for non-parametric continuous variables and categorical variables were compared using the maximum likelihood (ML) Chi-square test. Correlation between continuous variables was analysed by Spearman rank correlation test. Receiver operating characteristic (ROC) curve was used to determine a cut-off point of categorical predictors. Variables significant in univariate analysis were added to logistic regression to determine independent predictors of response to CRT. Stepwise variable selection with forward selection and backward elimination demonstrated identical results. Precision of the model was verified with the Hosmer-Lemeshow test of goodness of fit test. A *p* value < 0.05 was considered statistically significant.

## Results

Baseline characteristics of the subjects are summarized in Table 1. A total of 82 consecutive patients were included in the study. The study population consisted of 65 men (79.3%) and 17 women (20.7%), mean age 63.5 ± 10.5 years. ICMP related HF was diagnosed in 37 (45.1%) patients. Most of the patients (82.9%) were in NYHA class III. Mean 6-MWT was 300.8 ± 70.4 m. CRT and defibrillator (CRT-D) were implanted in 36 (43.9%) patients, twenty-five of them had developed paroxysmal monomorphic ventricular tachycardia (VT) before implantation. According to inclusion criteria all patients had a wide QRS complex (174.8 ± 17.0 ms), sinus rhythm, LBBB configuration and were treated according to HF guidelines [7], including beta-blockers, angiotensin converting enzyme inhibitors (ACE-I) or angiotensin receptor blockers (ARB), mineralocorticoid receptor antagonists (MRA), and diuretics at maximum tolerated doses.

**Table 1:** Baseline characteristics

**Patient characteristics**	**Baseline (n = 82)**
Age (yrs.)	63.5 ± 10.5
Gender (male,%)	65 (79.3)
QRS duration (ms)	174.8 ± 17.0
NYHA class III n (%)	68 (82.9)
Six minute walk test (m)	300.8 ± 70.4
Diabetes n (%)	9 (10.8)
Hypertension n (%)	66 (80.5)
Paroxysmal AF n (%)	28 (34.5)
VT n (%)	25 (30.5)
CRT-D n (%)	36 (43.9)
LVEF (%)	20.3 ± 6.5
LVEDD (mm)	68.5 ± 9.7
LVESD (mm)	61.9 ± 10.0
LVEDV (ml)	220.6 ± 76.3
LVESV (ml)	175.3 ± 69.8
LAV (ml)	93.3 ± 28.5
Beta blockers n (%)	77 (93.9)
ACE-I n (%)	61 (74.4)
ARB n (%)	16 (19.5)
MRA n (%)	70 (85.4)
Diuretics n (%)	68 (82.9)
Amiodarone n (%)	24 (29.3)
Statin n (%)	40 (48.8)
Aspirin n (%)	29 (35.4)
Warfarin n (%)	27 (32.9)
Uric acid (μmol/l)	426.3 ± 133.2

NYHA - New York Heart Association, AF – atrial fibrillation, VT- ventricular tachycardia, CRT-D – cardiac resynchronization therapy and defibrillator, LVEF – left ventricular ejection fraction, LVEDD – left ventricular end-diastolic diameter, LVESD - left ventricular end-systolic diameter, LVEDV - left ventricular end-diastolic volume, LVESV - left ventricular end-systolic volume, LAV – left atrial volume; ACE-I – angiotensin converting enzyme inhibitors; ARB - angiotensin receptor blockers; MRA - mineralocorticoid receptor antagonists.

**Figure 1:**
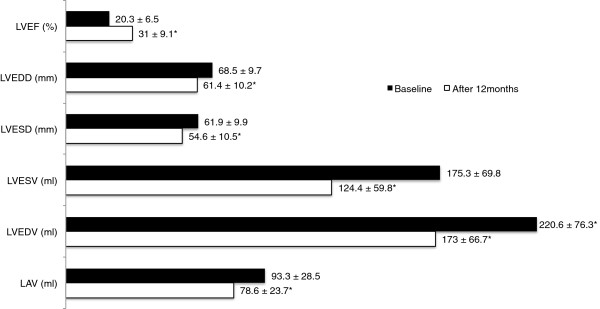
**Change in echocardiographic parameters during 12 months post CRT implantation (n = 76).** *p < 0.001 between the same initial and 12 months follow up echocardiographic parameter. LVEF – left ventricular ejection fraction, LVEDD – left ventricular end-diastolic diameter, LVESD - left ventricular end-systolic diameter, LVEDV - left ventricular end-diastolic volume, LVESV - left ventricular end-systolic volume, LAV – left atrial volume.

At 12 months of follow-up, a significant increase in LVEF (mean 10.4 ± 7.6%, p < 0.001), significant reduction in LV diameters (LVEDD -10.7 ± 16.5 mm, p < 0.001, LVESD -6.7 ± 7.0, p < 0.001), LV volumes (LVEDV -47.4 ± 53.7 ml, p < 0.001; LVESV -48.1 ± 50.2 ml, p < 0.001) and left atrial volume (LAV) (-14.0 ml ± 19.0, p < 0.001) were attained (Figure 1). In addition, a significant increase in 6-MWT (from 300.8 ± 70.4 m to 405.5 ± 65.7 m; p < 0.001) and decrease in QRS duration (from 174.8 ± 17.0 ms to 137.2 ± 15.0 ms; p = 0.001) were observed.

Six patients died within 12 months of CRT implantation. Due to the lack of full 12 month follow-up assessment, data of these patients were not included into the analyses of CRT response.

### Clinical response

**Figure 2:**
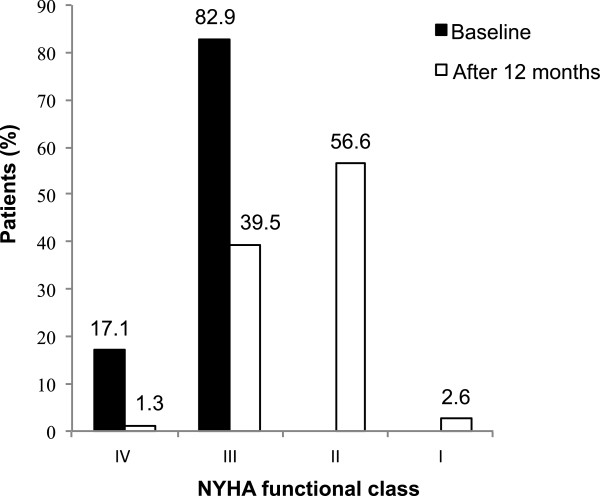
**Distribution of NYHA functional classes at baseline and after 12 months of CRT implantation (p = 0.001).** NYHA - New York Heart Association.

At 12 months follow-up 54 (71.1%) of patients had a significant improvement in NYHA class (p < 0.001). Distribution of NYHA class at baseline and after 12 months post CRT implantation is provided in Figure 2. An increase in 6-MWT by ≥ 15% was observed in 57 (75%) patients (p = 0.001). Mean increase in 6-MWT post CRT was 121.2 ± 66.1 m in clinical responders and 11.3 ± 27 m in non-responders (p = 0.001). Combined clinical response (improvement in NYHA class ≥ 1 class and/or ≥ 15% increase in the 6-MWT) was achieved in 63 (82.9%) patients (Table 2).

**Table 2:** Clinical and echocardiographic response to CRT at 12 months of follow-up

	**After 12 months (n = 76)**
Combined echocardiographic response	62 (81.6%)
LVEF increase ≥ 5%	62 (81.6%)
LVESV decrease ≥ 15%	56 (73.7%)
LVEDV decrease ≥15%	55 (72.4%)
Combined clinical response	63 (82.9%)
NYHA improvement ≥ 1 functional class	54 (71.1%)
Six minute walk test ≥ 15%	57 (75%)

NYHA - New York Heart Association, LVEF – left ventricular ejection fraction, LVEDV - left ventricular end-diastolic volume, LVESV - left ventricular end-systolic volume.

Compared to responders, non-responders were more likely to have ischemic cardiomyopathy (63.5% vs 36.5%, p = 0.01). Echocardiographic response was observed in 87.3% combined clinical responders (p = 0.03).

### Echocardiographic response

At 12 months of follow-up, LVEF increase of ≥ 5% was observed in 81.6% patients. Increase in LVEF was higher in patients with non-ICMP (11.2 ± 8.0 vs 7.7 ± 7.1; p = 0.04). Combined echocardiographic response (LVEF increase ≥ 5%, and/or LVESV decrease ≥ 15% and/or LVEDV decrease ≥15%) was established in 81.6% of the overall study population (Table 2). Compared to responders, non-responders were more likely to have lower LVEF, larger LVEDD, LVESD diameters and LV and LA volumes, AF and VT episodes at baseline, although only LV diameters, LAV, AF, VT achieved statistical significance (Table 3). Also, a negative association of warfarin use and echocardiographic response was found (p = 0.01). No significant differences in QRS duration, gender, age and CRT type between echocardiographic responders and non-responders were found.

**Table 3:** Comparison of echocardiographic and clinical parameters between combined echocardiographic responders and non-responders at baseline

	**Responders (n = 62)**	**Non-responders (n = 14)**	***p *value**
LVEF (%)	21.2 ± 6.5	17.9 ± 5.4	0.09
LVEDD (mm)	66.8 ± 9.8	75.3 ± 6.5	**0.01**
LVESD (mm)	60.0 ± 10.0	68.4 ± 7.8	**0.01**
LVEDV (ml)	211.0 ± 77.6	255.4 ± 50.6	0.05
LVESV (ml)	166.4 ± 69.3	201 ± 53.5	0.09
LAV (ml)	89.1 ± 30.2	104.5 ± 17.6	**0.02**
QRS duration (ms)	174.5 ± 16.6	175.7 ± 19.6	0.89
Six minute walk test (m)	305.6 ± 71.5	307.7 ± 57.3	0.72
Age (yrs.)	63.2 ± 10.1	60.2 ± 11.4	0.32
Gender (male,%)	51 (81)	9 (69.2)	0.34
Paroxysmal AF n (%)	18 (28.6)	8 (61.5)	**0.02**
VT n (%)	16 (25.4)	7 (53.9)	**0.04**
CRT-D n (%)	25 (39.7)	8 (61.5)	0.15
Beta blockers n (%)	61 (96.8)	12 (92.3)	0.45
ACE-I n (%)	48 (76.2)	9 (69.2)	0.59
ARB n (%)	13 (20.6)	2 (15.4)	0.66
MRA n (%)	52 (82.5)	12 (92.3)	0.38
Diuretics n (%)	51 (81)	13 (100)	0.08
Amiodarone n (%)	16 (25.4)	5 (38.5)	0.33
Statin n (%)	33 (52.4)	6 (46.2)	0.68
Aspirin n (%)	23 (36.5)	4 (30.8)	0.69
Warfarin n (%)	16 (25.4)	8 (61.5)	**0.01**
Uric acid (μmol/l)	397.7 ± 111.5	506.3 ± 159.0	**0.03**

LVEF – left ventricular ejection fraction, LVEDD – left ventricular end-diastolic diameter, LVESD - left ventricular end-systolic diameter, LVEDV - left ventricular end-diastolic volume, LVESV - left ventricular end-systolic volume, LAV – left atrial volume, AF – atrial fibrillation, VT- ventricular tachycardia, ACE-I – angiotensin converting enzyme inhibitors, ARB - angiotensin receptor blockers, MRA - mineralocorticoid receptor antagonists.

*p* value for the comparison between responders and non-responders.

Significant univariate predictors of favourable echocardiographic response after 12 months included smaller LVEDD (OR 0.89, 95% CI 0.82 - 0.97; p = 0.01) and LVESD (OR 0.91, 95% CI 0.85 - 0.98; p = 0.01). Lower uric acid concentration was associated with better echocardiographic response (OR 0.99, 95% CI 0.99 - 1.0; p = 0.01). The precision of the model was verified with the Hosmer-Lemeshow test of goodness of fit test (p = 0.1).

Non-ischemic HF etiology was an independent predictor of a positive clinical response (OR 4.89, 95% CI 1.39 - 17.15; p = 0.01). The precision of the model was verified with the Hosmer-Lemeshow test of goodness of fit test (p = 0.49).

**Figure 3:**
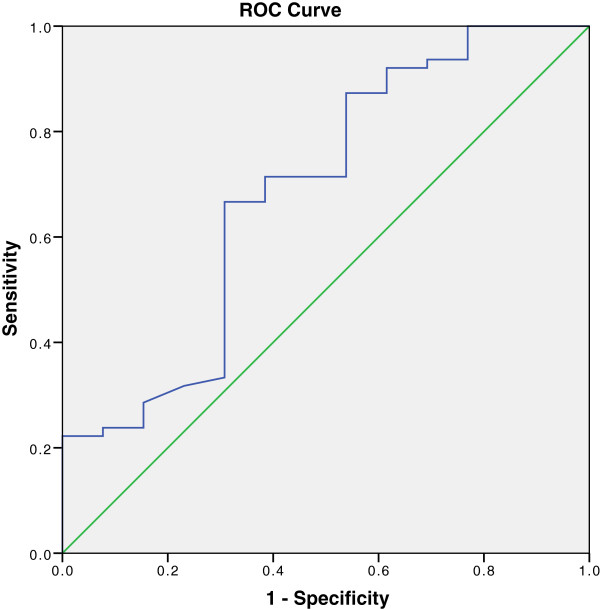
**ROC curve for the association of echocardiographic response and uric acid concentration.** (AUC 0.69, p = 0.03).

Only four parameters (Table 4) selected by regression analysis reached statistically significant cut-off values in ROC analyses, with sensitivity ranging from 59% to 81% and specificity from 61% to 77% (Figure 3).

**Table 4:** Echocardiographic and clinical parameters in the prediction of response to CRT

	**Area under curve (AUC)**	**95% CI**	**Sensitivity (%)**	**Specificity (%)**	**P value**
LVEDD < 75mm	0.77	0.64 - 0.89	81	62	0.03
LVESD < 64mm	0.74	0.6 - 0.88	70	69	0.01
LAV< 90ml	0.70	0.57 - 0.83	59	77	0.03
Uric acid < 440μmol/l	0.69	0.53 - 0.86	71	61	0.03

CI – confidence interval, LVEDD – left ventricular end-diastolic diameter, LVESD - left ventricular end-systolic diameter, LAV – left atrial volume.

Multiple stepwise regression analysis identified LVEDD of less than 75 mm (OR 5.60, 95% CI 1.36 - 18.61; p = 0.01) as the strongest independent predictor of favourable echocardiographic response, and non-ischemic HF etiology as the independent predictor of positive clinical response (OR 4.88, 95% CI 1.39 - 17.15; p = 0.01).

## Discussion

Prognosis of an HF patient depends on demographic, echocardiographic, haemodynamic, neurohormonal, and functional factors [8]. Each of these factors provided powerful independent prognostic information, yet they were poorly interrelated [9].

The present study confirmed that most patients treated with CRT demonstrated favourable clinical and echocardiographic responses during 12 months follow-up period. The baseline LV diameters, uric acid concentration and ischemic etiology were the main predictors of response to CRT.

Echocardiographic response was related to outcomes, but it was not associated with symptoms or quality of life. Dominique Auger et al. [10] reported NYHA improvement in ≥ 1 functional class in 80%, and combined clinical response in 84% of patients. Similar results were obtained in a study by Viviane Tiemi et al. [11], demonstrating improvement in NYHA functional class in 79% of patients after six months of CRT implantation. The 6-MWT was used as a measure of response to CRT in a number of studies, and was more sensitive compared to NYHA functional class [12]. Our data did not contradict with the findings of previous studies, as almost 82% of patients achieved significant improvement in clinical and echocardiographic parameters. The need for complicated assessment of CRT response is debatable, because from a patient’s perspective improvement in clinical condition matters most, hence changes in NYHA functional class and six-minute walk distance may be unsophisticated and important criteria for evaluation of response to CRT.

Previous studies [13,14] demonstrated that patients with ischemic heart disease had a lower likelihood of response to CRT. Gasparini et al. [15] reported that patients in the non-coronary artery disease (CAD) group had a significantly greater increase in LVEF (p = 0.007) and decrease in NYHA class (p < 0.05). Non-CAD patients had a greater increase in LVEF and decrease in NYHA functional class compared to patients with CAD. Sylvain Reuter et al. [16] explained differences of response according to etiology of HF, suggesting that left ventricular pacing lead was not placed at the optimal site with regard to ischemic areas. In contrast to our data and previously discussed studies, Molhoek et al. [17] reported no differences in CRT response in ischemic HF vs idiopathic dilated cardiomyopathy groups. However, in this study response to CRT was defined only by improvement in NYHA functional class. In our study patients with non-ICMP achieved a better clinical response compared to ischemic patients (63.9% vs 36.1%, p = 0.009).

In this study, receiver-operating characteristic curve analysis defined the optimal cut-off value of 64 mm for LVESD and 75 mm for LVEDD to predict the response to CRT, confirming previous analyses, [13,18] suggesting the larger cardiac diameters are associated with poorer response to CRT.

LA dilatation is a sensitive marker of chronic left heart disease. Pressure and/or volume overload associated with left ventricular involvement lead to gradual LA enlargement, electrical remodelling, and fibrosis. Considerable evidence has been collected, demonstrating relationship between increased LA size and cardiovascular morbidity and mortality [19-21] in general population [22] and in patients with left ventricular dysfunction [23,24]. In our study, baseline LA volumes were substantially lower in responders than in non-responders (89.1 ± 30.2 ml/m^2^ vs 104.5 ± 17.6 ml/m^2^, p = 0.02), and added to the prediction of response to CRT. More research is needed to analyse association of LA dilatation and response to CRT.

Higher uric acid concentration is associated with increased risk of mortality and morbidity in patients with HF [25,26]. Importance of uric acid in risk stratification is recognized by the Seattle Heart Failure Model. In our study, uric acid concentration was associated with response to CRT, with possible multifactorial mechanisms, i.e. increased xanthine oxidase activity induced oxidative stress and inflammation, and renal dysfunction related to hypoperfusion and diuretic therapy [25]. Further detailed studies are needed to define the exact mechanism of the association of increased uric acid and response to CRT.

In our study, there were no statistically significant differences in medication use at baseline between echocardiographic responders and non-responders, except in warfarin use (p = 0.01). Indirect influence of warfarin to the response to CRT might be explained by the AF incidence (non-responders 61.5% vs responders 28.5%), or by larger left atrium and ventricular diameters in non-responders.

Limitations of our study included a relatively small sample size, single centre participation and short study duration. Also, only patients with sinus rhythm were enrolled, the proportions of genders in the study were not equal.

## Conclusions

Smaller left ventricular end diastolic and end systolic diameters and lower serum uric acid concentration were associated with better response to CRT. LVEDD and non-ischemic HF etiology were the strongest independent predictors of positive response to CRT.

## Competing interests

The authors declare that they have no competing interest.

## Authors’ contributions

Study design: DR, SB, RJ. Data collection: DR, JL, KBD, KC, SA. Writing the first draft: DR. Data interpretation, discussion and preparation of the final manuscript: DR, SB, RJ, VZ, TK, VS, AP. All authors read and approved the final manuscript.

## Pre-publication history

The pre-publication history for this paper can be accessed here:

http://www.biomedcentral.com/1471-2261/14/55/prepub
